# Overexpression of MiR-29b-3p Inhibits Atrial Remodeling in Rats by Targeting PDGF-B Signaling Pathway

**DOI:** 10.1155/2021/3763529

**Published:** 2021-01-13

**Authors:** Xiangwei Lv, Pan Lu, Yisen Hu, Tongtong Xu

**Affiliations:** ^1^Department of Cardiology, Affiliated Hospital of Guilin Medical University, Guilin, 541001 Guangxi Zhuang Autonomous Region, China; ^2^Department of Cardiology, First Affiliated Hospital of Guangxi Medical University, Nanning, 530021 Guangxi Zhuang Autonomous Region, China

## Abstract

**Purpose:**

Studies have found that microRNAs (miRNAs) are closely associated with atrial fibrillation, but their specific mechanism remains unclear. The purpose of this experiment is to explore the function of miR-29b-3p in regulating atrial remodeling by targeting PDGF-B signaling pathway and thereby also explore the potential mechanisms.

**Methods:**

We randomly divided twenty-four rats into four groups. Caudal intravenous injections of angiotensin-II (Ang-II) were administered to establish atrial fibrosis models. Expressions of miR-29b-3p and PDGF-B were then tested via RT-PCR, western blot, and immunohistochemistry. Binding sites were then analyzed via the bioinformatics online software TargetScan and verified by Luciferase Reporter. We used Masson staining to detect the degree of atrial fibrosis, while immunofluorescence and western blot were used to detect the expressions of Collagen-I and a-SMA. We used immunohistochemistry and western blot to detect the expression of connexin 43 (Cx43).

**Results:**

In comparison with the Ang-II group, miR-29b-3p was seen to lower the degree of atrial fibrosis, decrease the expression of fibrosis markers such as Collagen-I and a-SMA, and increase the protein expression of Cx43. MiR-29b-3p can lower the expression of PDGF-B, while the Luciferase Reporter showed that PDGF-B is the verified target gene of miR-29b-3p.

**Conclusions:**

MiR-29b-3p was able to reduce atrial structural and electrical remodeling in the study's rat fibrosis model. This biological function may be expressed through the targeted regulation of the PDGF-B signaling pathway.

## 1. Introduction

The prevalence of atrial fibrillation (AF) increases with age and concomitant heart diseases. Due to severe complications, such as embolic diseases and heart failure, the incidence and mortality rates associated with cardiovascular and cerebrovascular diseases have increased significantly [1–3]. Although currently, there are many AF treatment options, their therapeutic effects still present challenges, and their potential side effects cannot be ignored. Subsequently, studying new mechanisms associated with AF is critical towards finding new, more efficacious treatment methods.

The pathophysiological mechanisms of AF are extremely complex, and its specific pathogenesis has yet to be fully understood, but atrial structural remodeling and electrical remodeling have been proven to contribute to the onset and maintenance of AF [4]. Atrial fibrosis plays a critical function in atrial structural remodeling and is a signature change of such remodeling. Studies have confirmed that atrial fibrosis is caused by the interaction between atrial cardiomyocytes and fibroblasts and that their interaction can cause arrhythmia changes due to the bioelectricity of the cardiomyocytes [5]. Other studies have shown that atrial electrical remodeling is also believed to play an important function in the onset and maintenance of AF [6, 7]. Atrial electrical remodeling includes both ion channel remodeling and intercellular gap remodeling, which refers to the abnormal expression and distribution of gap junctional proteins (GJPs). Connexin 43(Cx43) is the primary GJP in myocardial tissue and plays an important function in the electrical connection and conduction between myocardial cells. As such, it has been shown that abnormal expression of Cx43 can cause the onset and maintenance of AF [8–10].

Platelet-derived growth factors (PDGF) include the four isotypes of PDGF-A, PDGF-B, PDGF-C, and PDGF-D, which are composed as various dimers, namely, PDGF-AA, PDGF-BB, PDGF-CC, PDGF-DD, and PDGF-AB. PDGF exercise their biological activity by binding with receptors PDGFR-*α* and PDGFR-*β* of cell membranes [11]. Recent studies have found that the PDGF-B (PDGF-BB) signaling pathway has regulatory effects on myocardial fibrosis and Cx43 expression, thereby playing an important role in the onset and development of AF [12–14].

MicroRNAs (miRNAs) are a kind of noncoding RNA, which regulates the posttranscription level of the mRNA expression by binding to the 3′Untranslated Region (3′-UTR) of target gene mRNA [15]. Recent studies have discovered that miRNAs can promote the onset of atrial remodeling. As such, they play an important role in the onset and development of AF and can provide an important evidence for the clinical diagnosis and treatment of AF [16–20]. Studies have discovered that the miR-29 family plays an important function in the onset of myocardial fibrosis through their regulation of related target genes [21–24]. However, the specific mechanisms involved have not been fully elucidated. In this study, our aim is to investigate whether one member of the miR-29 family, miR-29b-3p, inhibits Ang-II induced atrial remodeling in rats through PDGF-B signaling pathway.

## 2. Materials and Methods

### 2.1. Animals and Theory

The animal subjects of this study were Sprague-Dawley rats, who were male, 8 weeks old, and weighing 220-250 g. All experimental procedures and protocols were performed under the guidance of the Ethic Commission of Experimental Animals of Guangxi Medical University. The laboratory animals were raised under standard experimental conditions: humidity 50%-60%, temperature 20°C-25°C, and 12-hour alternating light and dark cycles, with clean drinking water and feed supplied throughout their day. Before and after caudal intravenous injections were administered, 75% medical alcohol was applied for sterilization and to avoid infection. The animal subjects were euthanized under anesthesia to minimize pain as much as possible.

### 2.2. Construction of Disease Model and Transfection of Adeno-Associated Virus

Twenty-four rat subjects were randomly put into four groups: Sham group, Ang-II group, AAV+NC group, and AAV+miR-29b-3p group. The recombinant adeno-associated virus (serotype 9) was used as vector, AAV2/9-cTNT-GFP (AAV+NC) as the negative control group, AAV2/9-cTNT-miR-29b-3p-GFP (AAV+miR-29b-3p) overexpressed miR-29b-3p, and c-TNT was used as promoter in specific expression in myocardial tissue. The adeno-related virus was designed and quality tested by Hanbio Biotechology (Shanghai, China) and used in the experiment according to instructions. The four groups were administered the following respective treatments:

1. Sham group: 0.9% normal saline 2 ml/kg/day via caudal intravenous injection for 14 days. (2) Ang-II group: Ang-II (2 mg/kg/day, Solarbio, China) via caudal intravenous injection for 14 days. (3) AAV+NC group: AAV-NC (2 × 10^11^ vector genomes (vg) particles/per rat) adeno-associated virus was transfected via a singular caudal intravenous injection. 14 days after the transfection, Ang-II (2 mg/kg/day) was administered via caudal intravenous injection for 14 days. (4) AAV+miR-29b-3p group: adeno-associated virus, AAV+miR-29b-3p (2 × 10^11^ vector genomes (vg) particles/per rat) was transfected via a singular caudal intravenous injection. 14 days after the transfection, Ang-II (2 mg/kg/day) was administered via caudal intravenous injection for 14 days. Following the experiment, pentobarbital sodium (40 mg/kg) was injected via intraperitoneal into the rats for anesthesia to harvest their hearts

### 2.3. Real-Time PCR (RT-PCR) Detection

Following anesthesia, the heart was quickly removed, and the atrial tissue was isolated. 1 ml of TRIzol Reagent was added to 100 mg myocardial tissue, grounded thoroughly, followed by being centrifuged at 12,000 rpm for 10 minutes. The resulting supernatant was then taken. NanoDrop 2000 spectrophotometer was used to test the samples' total RNA concentration and purity according to its operating instructions. RNA with too high of a concentration was diluted proportionally to make its final concentration 200 ng/*μ*l. The level of miR-29b-3p was then detected via a TaqMan Micro Assay Kit (Thermo Fisher Scientific, USA) according to the manufacturer's instructions. RT-PCR detection and data analysis were performed using the ABI 7500 RT-PCR System (Applied Biosystems, USA) and Fast SYBR Green Master Mix Kit (Applied Biosystems, USA) according to the operating instructions. The primers used in the experiment are as follows: miR-29b-3p forward, 5′-ACACTCCAGCTGGGTAGCACCATTTGAAA-3′ and reverse, 5′-CTCAACTGGTGTCGTGGAGTCGGCAATTCAGTTGAGAACACTGA-3′; PDGFB forward, 5′-TCCGCTCCTTTGATGACCTT-3′ and reverse, 5′-TCCGACTCGACTCCAGAATGT-3′; U6 forward, 5′-CTCGCTTCGGCAGCACA-3′ and reverse, 5′-AACGCTTCACGAATTTGCGT-3′; and *β*-actin forward, 5′-TGCTATGTTGCCCTAGACTTCG-3′ and reverse, 5′-GTTGGCATAGAGGTCTTTACGG-3′. The primers used in the experiment were designed by Sangon Biotech (Shanghai, China). Fold changes in the miR-29b-3p and PDGF-B expression were calculated using 2^-△△Ct^ method. U6 or *β*-actin was used as the internal control, respectively, in the experiment.

### 2.4. Target Gene Prediction and Luciferase Reporter Testing

**Figure 1:**
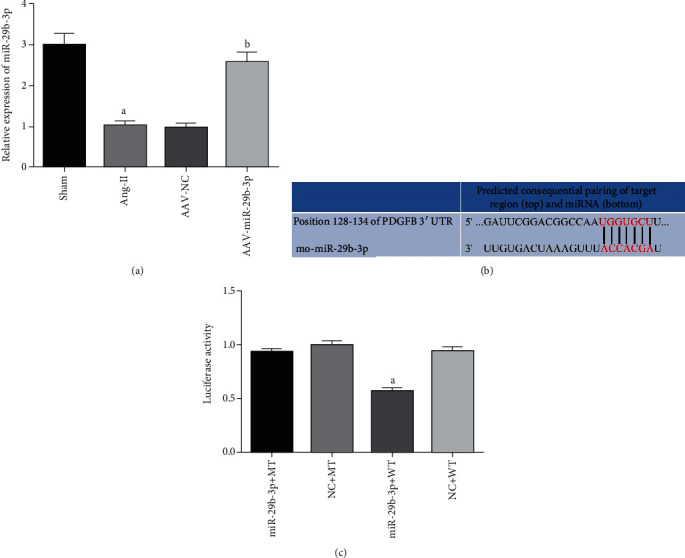
MiR-29b-3p expression levels and its stable binding with PDGF-B. (a) RT-PCR detects the expression of miR-29b-3p in atrial tissue. (b) TargetScan's predicted binding sites of miR-29b-3p and PDGF-B. (c) Luciferase Reporter testing results. These results are statistically analyzed using the Prism 6.0 software. Note that ^a^*P* < 0.05 compared with the Sham group or the miR-29b-3p+MT group; ^b^*P* < 0.05 compared with the Ang-II group.

As shown in Figure 1(b), the bioinformatics online software TargetScan was used to forecast the binding sites of miR-29b-3p and PDGF-B. A Luciferase Reporter was then used to verify whether PDGF-B combined with miR-29b-3p at these sites. Wild-type (WT) and mutant-type (MT) PDGF-B 3′-UTR (Wilking, China) were constructed. Using HEK 293T cells in a logarithmic growth phase, 2 × 10^4^ cells per well were inoculated into 96-well plates and then cultured. Synthesized miR-29b-3p mimics or negative-control (NC) (GenePharma, China) were transfected for 48 hours according to Lipofectamine®3000 Transfection Reagent's operating instructions. Luciferase activity was then detected via a Dual-Luciferase Reporter System (Promega, USA).

### 2.5. Masson Staining Analysis

The rats were anesthetized with pentobarbital sodium (40 mg/kg) via intraperitoneal injection, and their hearts were quickly taken out under anesthesia. Postremoval, blood was washed with 0.9% normal saline, and 4% paraformaldehyde is used to fix the atrial tissue. 3 mm thick slices were dewaxed, and Masson staining was performed according to operating instructions. An optical microscope (Olympus, Japan) was used to observe the samples, and three ×200 magnifications were randomly selected from each sample.

### 2.6. Immunohistochemistry and Immunofluorescence Detection

The 3 mm thick slices were taken and incubated overnight at 4°C with PDGF-B and Cx43 primary antibodies, with a dilution concentration of 1 : 150. Following these primary antibodies incubation, the samples were washed for 5 minutes and then incubated with secondary antibodies at room temperature for 4 hours. Then, an optical microscope (Olympus, Japan) was used to observe the samples, and three ×200 magnifications were randomly selected from each sample. We adopted an immunofluorescence approach to detect the expression of Collagen-I in the atrial tissue. The prepared samples were sealed within 10% serum for 2 hours, then incubated overnight at 4°C with Collagen-I primary antibodies, with a dilution concentration of 1 : 150, and colocalization testing was performed. The samples were then washed and incubated with secondary antibodies at room temperature for 4 hours. DAPI staining was done for 10 min before observation via an optical microscope (Olympus, Japan), with three ×200 magnifications randomly selected from each sample.

### 2.7. Western Blot Analysis

For each experimental group, 100 mg of atrial tissue was placed into a RIPA buffer solution of phenylmethylsulfonyl fluoride (PMSF) to fully lyse the tissue. Protein concentrations were tested via BCA assay kits (Beyotime, China), analyzed using sodium dodecyl sulfate-polyacrylamide gel electrophoresis (SDS-PAGE), and then transferred to a polyvinylidene fluoride (PVDF) membrane. At room temperature, the PVDF membranes were incubated by PDGF-B (1 : 1000, Abcam), Collagen-I (1 : 2000, Abcam), a-SMA (1 : 1000, CST), Cx43 (1 : 1000, CST), GAPDH (1: 1000, CST), and *β*-tubulin (1 : 1000, CST) for 6 hours. The PVDF were then incubated with secondary antibodies (1 : 14000, Beyotime) at room temperature for 4 hours. Signals were detected via a Chemiluminescence System (Amersham Pharmacia).

### 2.8. Data Statistics

This study's statistical data was expressed using average values ± standard deviations, while Student's *t*-test or one-way ANOVA was used for data analysis, and Prism 6.0 was used for statistical analysis. *P* values less than 0.05 are believed to indicate statistical significance. Pathological experiment results were analyzed by the Image-Pro Plus 6.0 software.

## 3. Results

### 3.1. MiR-29b-3p Expression Levels and Target Gene PDGF-B Verification

RT-PCR detection, as shown in Figure 1(a), miR-29b-3p decreased in the An-II group in comparison with the Sham group. Overexpression of miR-29b-3p (as in the AAV-miR-29b-3p group) can increase the level of miR-29b-3p in atrial tissue compared with the Ang-II group. In order to further observe the biological function of miR-29b-3p, a target gene was predicted by TargetScan bioinformatics online software, and this suggested that PDGF-B is its potential target gene. The subsequent Luciferase Reporter testing showed that the expression of WT miR-29b-3p decreased in comparison with MT miR-29b-3p. This further suggested that PDGF-B is a target gene of miR-29b-3p and that miR-29b-3p has a significant inhibitory effect on it, as shown in Figures 1(b) and 1(c).

### 3.2. MiR-29b-3p Can Inhibit the Expression of the PDGF-B Signaling Pathway

**Figure 2:**
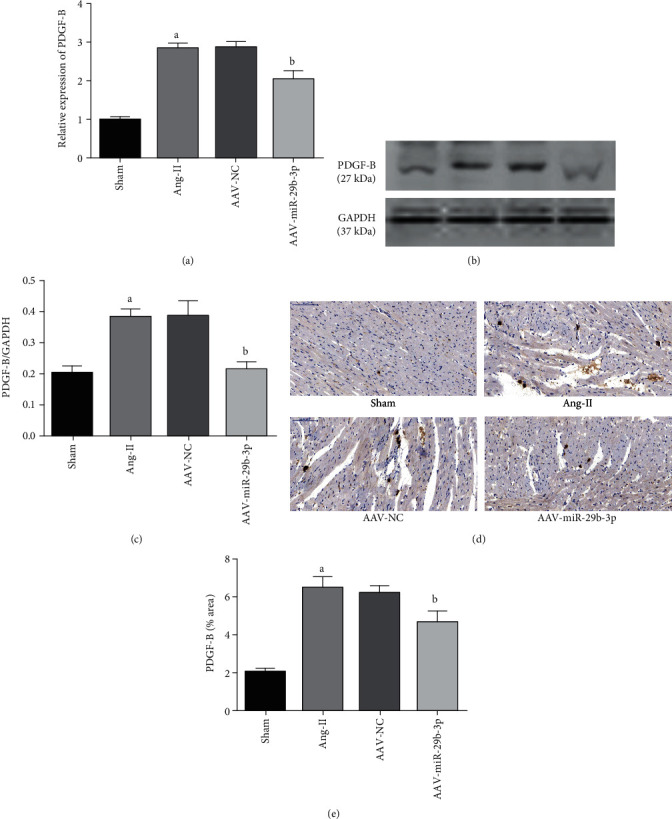
MiR-29b-3p can inhibit PDGF-B expression. (a) RT-PCR detection results. (b, c) Western blotting testing and PDGF-B expression and quantitative analysis. (d, e) Immunohistochemical testing (×200, 100 *μ*m) results and quantitative analysis. These results are statistically analyzed using the Prism 6.0 and Image-Pro 6.0 software. Note that ^a^*P* < 0.05 compared with the Sham group; ^b^*P* < 0.05 compared with the Ang-II group.

RT-PCR detection showed that in comparison with the Sham group, the Ang-II group's expression level of PDGF-B in atrial tissue was significantly increased, while with an overexpression of miR-29b-3p, the AAV-miR-29b-3p group had a reduced PDGF-B expression level compared with the Ang-II group (Figure 2(a)). Similarly, western blot and immunohistochemistry testing proved that the overexpression of miR-29b-3p (AAV-miR-29b-3p group) appeared to inhibit the PDGF-B expression compared with the Ang-II group (Figures 2(b)–2(e)).

### 3.3. MiR-29b-3p Can Reduce the Degree of Atrial Fibrosis

**Figure 3:**
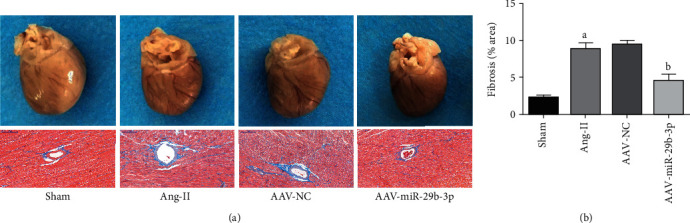
MiR-29b-3p can lower the degree of atrial fibrosis. (a) Overview observation of heart sample and Masson staining (×200, 100 *μ*m) and analysis (b). These results are statistically analyzed using the Prism 6.0 and Image-Pro 6.0 software. Note that ^a^*P* < 0.05 compared with the Sham group; ^b^*P* < 0.05 compared with the Ang-II group.

Overall visual observations discovered that in comparison with the Sham group, the cardiac blood vessels in the Ang-II group were significantly dilated and congested, and the degree of fibrosis of the perivascular tissues increased (Figure 3(a)). However, compared with the Ang-II group, overexpression of miR-29b-3p in the AAV-miR-29b-3p group appeared to lower the degree of cardiac vasodilation and congestion, while simultaneously reducing the degree of fibrosis (Figures 3(a) and 3(b)).

### 3.4. MiR-29b-3p Can Reduce the Expression Level of Fibrosis Markers

**Figure 4:**
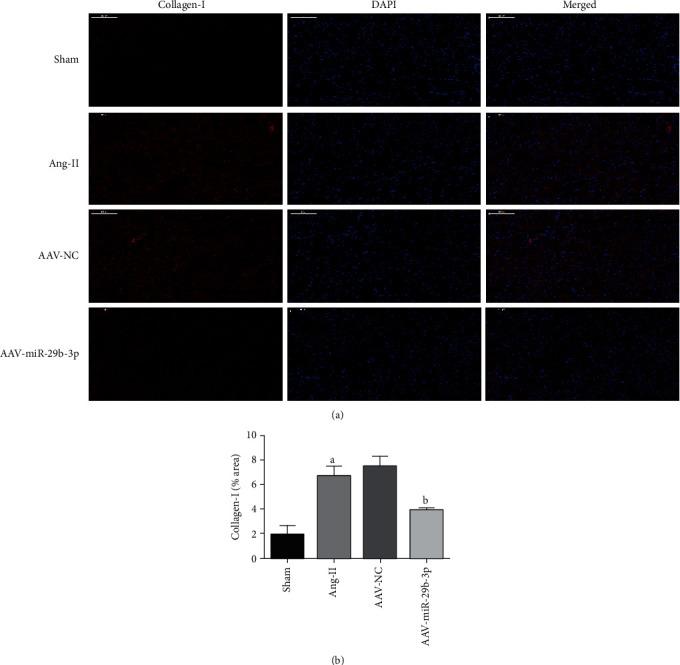
MiR-29b-3p can reduce the expression level of fibrosis markers. (a) Immunofluorescence testing (×200, 100 *μ*m) results: red light represents Collagen-I expression; blue light represents total myocardial nucleus (b) quantitative analysis. These results are statistically analyzed using the Prism 6.0 and Image-Pro 6.0 software. Note that ^a^*P* < 0.05 compared with the Sham group; ^b^*P* < 0.05 compared with the Ang-II group.

**Figure 5:**
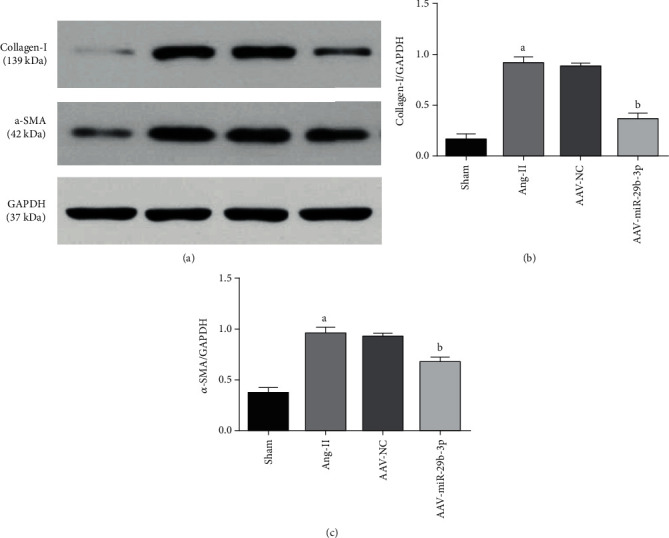
MiR-29b-3p can reduce the expression of fibrosis markers. (a) Western blotting testing and fibrosis marker expression and quantitative analysis (b, c). These results are statistically analyzed using the Prism 6.0 and Image-Pro 6.0 software. Note that ^a^*P* < 0.05 compared with the Sham group; ^b^*P* < 0.05 compared with the Ang-II group.

1. Immunofluorescence testing discovered that the expression of Collagen-I within the Ang-II group was significantly higher than within the Sham group. Correspondingly, overexpression of miR-29b-3p within the AAV-miR-29b-3p group appeared to reduce the expression level of Collagen-I against the Ang-II group (Figures 4(a) and 4(b))

(2) Western blot testing also confirmed that the expression of the atrial fibrosis markers Collagen-I and a-SMA was significantly increased within the Ang-II group in comparison with the Sham group, while overexpression of miR-29b-3p within the AAV-miR-29b-3p group appeared to reduce their expression against the Ang-II group (Figures 5(a) and 5(b))

### 3.5. MiR-29b-3p Can Increase the Expression of Cx43 in Atrial Tissue

**Figure 6:**
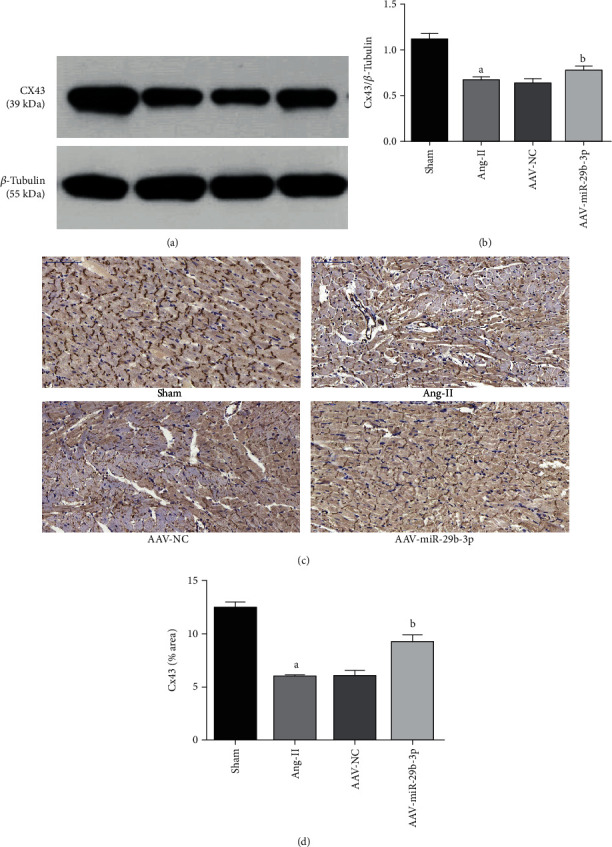
MiR-29b-3p can increase the expression of Cx43. (a, b) Western blotting testing and Cx43 expression and quantitative analysis. (c, d) Immunohistochemical testing (×200, 100 *μ*m) results and quantitative analysis. These results are statistically analyzed using the Prism 6.0 and Image-Pro 6.0 software. Note that ^a^*P* < 0.05 compared with the Sham group; ^b^*P* < 0.05 compared with the Ang-II group.

Western blot and immunohistochemistry testing indicated that the expression of Cx43 within the Ang-II group was lowered compared with the Sham group. Inversely, overexpression of miR-29b-3p within the AAV-miR-29b-3p group appeared to increase the expression of Cx43 compared with the Ang-II group (Figures 6(a)–6(d)).

## 4. Discussion

In this study, we found that Ang-II can induce the low expression of miR-29b-3p in the atrial tissue of rats, increase the degree of atrial fibrosis, and decrease the expression of Cx43, while also corresponding to a significant increase in the expression of PDGF-B. Subsequently, through the overexpression of miR-29b-3p triggered by an adeno-associated virus, we discovered that the overexpression of miR-29b-3p can reduce the degree of atrial fibrosis, increase the protein expression of Cx43, and simultaneously significantly reduce the expression of PDGF-B. Additionally, our analysis via the bioinformatics online software TargetScan showed that PDGF-B is a potential target gene of miR-29b-3p, and the subsequent Luciferase Reporter testing confirmed that there is stable binding between PDGF-B and miR-29b-3p. Our results strongly indicated that in HEK 293T cells, miR-29b-3p can negatively adjust the expression of PDGF-B.

### 4.1. MiR-29b-3p Is Participated in the Onset of AF through Regulating Atrial Structural Remodeling

Through the pathological process of AF occurrence and development, structural remodeling is a common pathological feature, and fibrosis is the signature change of structural remodeling. A large number of studies have demonstrated that miRNAs play an important function in AF occurrence by regulating atrial fibrosis and structural remodeling [25, 26]. Through studies of rabbit AF model, some researchers have found that miR-21 inhibits the posttranscription of Smad7, improves the activity of TGF-*β*1/Smad pathway, and is involved in the occurrence of atrial fibrosis [27]. Other researchers have found through rat AF model studies that the downregulation of miR-10a may reduce atrial fibrosis and structural remodeling by inhibiting the expression of TGF-*β*1/Smads signaling pathway [28]. Still other studies of patients with nonvalvular paroxysmal AF and the primary cardiac fibroblasts of C57BL6 mice have found that miR-146b-5p is involved in AF occurrence by targeting TIMP-4 to regulate atrial fibrosis [29].

In this study, we discovered that overexpression of miR-29b-3p can reduce the degree of Ang-II induced atrial fibrosis in rats and reduce the expression levels of Collagen-I and a-SMA. Therefore, we cautiously believe that miR-29b-3p may participate in AF occurrence by regulating atrial fibrosis and structural remodeling.

### 4.2. MiR-29b-3p Plays an Important Function in AF Onset by Regulating Atrial Electrical Remodeling

Atrial electrical remodeling also plays a key function in the pathological process of AF. Atrial electrical remodeling leads to a shortening of the effective refractory period, an increase of triggered activities, and an increase in the dispersion and slowing down of the conduction velocity of atrial cardiomyocytes, which is conducive to the formation of local microreentry loops and plays an important function in the onset and maintenance of AF [30, 31]. A characteristic of electrical remodeling is the change in GJP function and expression, especially that of Cx43, which is closely related to AF occurrence [32]. Abundant clinical and animal studies have shown that miRNAs also play an important function in AF occurrence by regulating atrial electrical remodeling [33]. Through studies of AF patients with valvular disease, as well as dog and mice models, some researchers have found that miR-328 is involved in AF occurrence by targeting CACNA1C and CACNB1 to regulate atrial electrical remodeling [34]. Other researchers studying AF patients and HL-1 cell models have found that miR-499 regulates atrial electrical remodeling by targeting SK3, which also plays an important role in AF occurrence [35]. Additionally, through the study of AF patients, sheep and HL-1 cell models, other scholars have found that miR-208b is involved in the onset of AF by targeting CACNA1C and CACNB2 to regulate atrial electrical remodeling [36].

In this study, we found overexpression of miR-29b-3p can also increase the expression level of Cx43, and subsequently, we speculated that miR-29b-3p may also participate in AF occurrence by regulating electrical remodeling, indicating a new potential function of miR-29b-3p.

### 4.3. MiR-29b-3p Exerts a Biological Function by Targeting PDGF-B Signaling Pathway to Regulate Fibrosis and Cx43 Expression

PDGF is highly expressed throughout the process of cardiomyocyte growth and maturation and regulates physiological functions through stimulating the proliferation, differentiation, and migration of mesenchymal cells and myocardial fibroblasts [37–39]. PDGF-BB dimer is the most widely studied isotype at present, and it is the only growth factor that can bind to PDGFR-*α* and PDGFR-*β* receptors on cell membranes and trigger different signal cascades. As such, it is referred to as the universal isotype of PDGF [40].

Some researchers have found through mouse model studies that the PDGF-B signaling pathway is closely related to myocardial fibrosis. Mouse model studies have shown that myocardial infarction can activate the PDGF-BB/PDGFR-*β* signaling pathway, which leads to the formation of fibrosis and increased collagen content. Use of the PDGFR-*β* receptor blocker (APB5) can reduce the deposition of collagen in infarct sites and reduce myocardial fibrosis [41]. Meanwhile, some scholars have found through rat model studies that the PDGF-B signaling pathway can help alleviate ischemia/reperfusion injuries related to heart transplants by regulating myocardial fibrosis [42]. Through other studies on fibroblast models of AF patients, dogs, and rats, other researchers have discovered that the PDGF-BB/PDGFR-*β* signaling pathway promotes the proliferation of cardiac fibroblasts and the secretion of type I collagen, which is an element in the formation of atrial fibrosis and may become a new focus for AF intervention [12].

**Figure 7:**
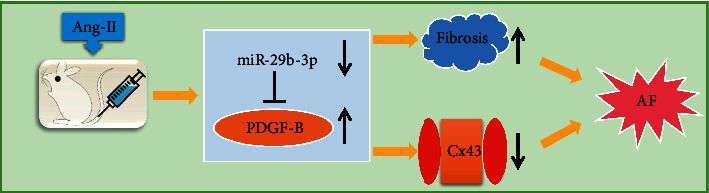
The molecular mechanism diagram shows that miR-29b-3p may participate in the onset of atrial fibrillation by the PDGF-B signaling pathway to regulate atrial remodeling.

In this study, we found that the expression of the PDGF-B signal pathway was significantly increased in the Ang-II induced rat model, while the overexpression of miR-29b-3p could reduce the expression level of PDGF-B. Subsequent Luciferase Reporter testing confirmed that PDGF-B is the direct target gene of miR-29b-3p. Therefore, we speculated that miR-29b-3p regulates atrial remodeling, possibly by targeting the PDGF-B signaling pathway, as summarized schematically in Figure 7.

## 5. Conclusion

In Ang-II induced rat atrial fibrosis model, we discovered that miR-29b-3p and PDGF-B showed significant changes in their expression levels. In the experiment, miR-29b-3p showed a significant antifibrosis effect via atrial structural remodeling, while also having a significant impact on the expression of Cx43 which is related atrial electrical remodeling. Based on these results, we tentatively speculate that miR-29b-3p may inhibit both atrial structural remodeling and electrical remodeling by targeting PDGF-B signaling pathway. This may represent the discovery of a new molecular mechanism of AF occurrence and a potential area for the disease's therapeutic research.

## Data Availability

The data used to support the findings of this study are available from the corresponding author upon request.
